# 

**Published:** 1845-03

**Authors:** 


					CON TENT 8.
161
-Contributions to Operative and Mechanical Dentistry.
I o:
mm
m ?;* IW* . M . . ??' ?? ??-*??? ?
'* '
p.
hen. Wheeling, Va.,
166
Article II.?
. * ?.??.,>??????..????... ?.? ? I
-Dentistry and Physie.
By the Syracuse Editor,
174
Article III.-
T&e Morbid Effects of Decayed Teeth and a Diseased
O. Cone, D. D. S.,dt Barnstable, Mass.
187
K : ~St:-
' ?**"
.>?.????-5'i ? rw -w-.-.?-
-An Essay on Odontalgia, its Causes and its 1 reatmeot. j
By. S. E. Arms, M. D.
pm?;???jssgg? ;ii?spsk. y^s?
??
-Osseous Union of Teeth.
By Jumes D. McCabe,
D. D. S., of Richmond, Va ,
224
Article flft'S-
m
-Dr. Somerby's Patent Blow-pipe and Furnace,
Article VII.-
COLLECTANEA.
Epilepsy cured by extracting a toreiga body irom the Meat
torious,
?
Letter to the Editor of the Southern Medical aod Surgieal Journal,..
"i. . - , 7 - "v
were extracted from the Wrist,
Liberia Medical School,
283
itures for the Insane,
???
Jefferson Medical College,
234
7 7- V- - , 7' - \ - 7 . - " ' ; " . ;
. . * .. ? . ...... ? ? ???? ? ? ? * ? ? '??
Rejection of Quack Advertisements,
? ? ?
Case of Catalepsy, with Cd*l*i|lsions Pi the Right Arm,
236
?
The Principles and Praetit# of Dental Surgery.
23G
By Cfcapin
Hams, M.Pt/0. D. S, ? ? ? * ? ????? * ? ? ft ? ? t ? ? ? # i ? t ? ? ? ? ? ? * ? * ?
A "Tatsasassa swatawsgi
By James Copeland, M. U& F. R. SQIEdited, with additions,
by Charles A. Lee, M. D. Part 4th  ,
Principles of Forensic Medicine.
m
24'2
By William A. Guy, M.B. V First
American edition. Wttfc-notes nnd additions, f By |Stgles A
u?i?m.m$
??-,V
Medical and Surgical Journal.
242
Edited by Paul F.
0., and J. P. Garvm, M. D ? J. ...... .... . ? ? . . . . . . . . . ? ?
New Orleans Medical Journal, devoted to the Cultivation of Medi-
cine and the Associate Sciences.
243
> M. D? and A, Hester, M. D.,..........? ?.??? |
? ? ???
New York Journal of Medicine,
243
-Vr.
> * ? ? ? *? > ??? ? ? ? ? ? ? ? ? ?_? ? ?>. ? ? ? ,
rj ?
cuw Hygiene,* ....... ? ? ? ? ? ? ?>? ? ? ? ??? ? *
iviorbid Dental Specimens,. .. 1:-.. $i..... ?...
Ossification of the Pulp of a Tooth,....; *
Blandin's Human and Comparative Dental Anatomy,.
Popular Treatise on the Teeth,.**...
? ???
Self-Education,.,.
Dental EUostosif^.1
A Threat Explained,. , i
Prussia,...
Virginia Society of Surgeon Dentists, I.........  247 .
I ; regulating the Practice of Dentistry in Alabama,.     0><fl
Cincinnati College of Dental Surgery,
Itinerant Dentist,
Baltimore College of Dental Surgery,...

				

